# First report of GI.1aP-GI.2 recombinants of rabbit hemorrhagic disease virus in domestic rabbits in China

**DOI:** 10.3389/fmicb.2023.1188380

**Published:** 2023-07-14

**Authors:** Yan Li, Deyan Du, Long Zhou, Liyin Su, Chengcheng You, Huai Zhang, Jifeng Yu, Lu Xiao, Jian Huang

**Affiliations:** ^1^College of Animal Science and Veterinary Medicine, Southwest Minzu University, Chengdu, China; ^2^Huapai Biological Group, Chengdu, China; ^3^Sichuan Animal Science Academy, Sichuan Provincial Key Laboratory of Animal Breeding and Genetics, Chengdu, China

**Keywords:** recombination, GI.1aP-GI.2 variants, phylogenetic features, rabbit hemorrhagic disease virus 2, pathogenicity

## Abstract

The rabbit hemorrhagic disease virus 2 (RHDV2 or GI.2) is a highly contagious agent leading to lethal disease in rabbits. It frequently recombines with other *Lagovirus* genus, generating epidemical variants with high pathogenicity. In this study, twenty-two liver samples tested positive for GI.2 VP60 gene, were collected in rabbit farms from several geographical regions in China. All GI.2 positive specimens were submitted for RT-PCR detection, nucleotide sequencing and phylogenetic analysis. In addition, suspected GI.2 recombinants were evaluated for virus virulence. The results showed that nine presumptive recombinants were identified by testing for RdRp-VP60 recombination. In these recombinants, four were selected to fully characterize the genome of novel GI.2 recombinant variants, which were described as GI.1aP-GI.2. The nucleotide sequence of these novel variants showed unique recombination pattern and phylogenetic features compared to currently prevalent GI.2 variants. Furthermore, this distinctive recombination of new variant SCNJ-2021 moderately enhanced the virulence of GI.2, even for rabbits vaccinated against parental GI.2. In conclusion, the novel GI.1aP-GI.2 recombinants were identified in rabbit industry in China for the first time, which expanded the knowledge on the phylodynamics and genomic diversity of GI.2 genotype. The rapid molecular evolution and varied pathogenicity of these virus recombinants highlight the urgent need for epidemiological surveillance and for future prevention of these neglected GI.2 variants.

## 1. Introduction

The rabbit hemorrhagic disease virus (RHDV) is a common and highly contagious agent that causes acute multi-organ hemorrhagic syndrome with high morbidity and mortality (Teifke et al., 2002; Neimanis et al., 2018a). RHDV is a positive-sense single-stranded RNA virus from the *Lagovirus* genus, *Caliciviridae* family (Lopes et al., 2017). Each particle contains a genome of about 7.4 kb and a subgenomic RNA (sgRNA) of about 2.5 kb. The genome encodes two open reading frames (ORFs) with slightly overlapping. ORF1 encodes a large polyprotein that is cleaved by a virus-encoded protease, generating seven non-structural proteins (NSPs) and the major structural protein (VP60), and ORF2 encodes a minor structural protein (VP10) (Le Pendu et al., 2017). Based on the phylogenetic classification and VP60 gene variability, the RHDVs were divided into GI.1 and GI.2 genotypes. The GI.1 genotype was further subdivided into classic RHDV (G1/GI.1b, G2/GI.1c and G3-G5/GI.1d) and antigenic variant RHDVa (G6/GI.1a) (Le Pendu et al., 2017).

Since the first outbreak in Jiangsu province, China, in 1984 (Liu et al., 1984), classic RHDVs that underwent constant evolution with cumulative genomic alterations presented varied etiologic and epidemiological features (Wang et al., 2012; Abrantes et al., 2020a). In the last twenty years, GI.1c and GI.1a genotypes co-circulated in China along with intergenotypic recombination during their widespread transmission (Hu et al., 2016, 2017). In 2010, a new RHDV variant was identified in France, named RHDVb or RHDV2 (GI.2), which showed distinctive genetic and antigenic characteristics compared to GI.1. Moreover, this virus exhibited low cross-protection with other *Lagoviruses* (Le Gall-Reculé et al., 2011). Subsequently, the highly pathogenic GI.2 damaged the rabbit industry heavily in Europe, Australia, Africa, and North America, which rapidly replaced GI.1 as the predominant genotype in the past decade (Dalton et al., 2012; Mahar et al., 2018; Neimanis et al., 2018b; Chehida et al., 2021; Aguayo-Adán et al., 2022). Owing to the rapid spread of RHDVs and the resulting economic and ecological losses, the emerging pathogenic GI.1a and GI.2 raised increased concern in recent years (Lopes et al., 2017; Pacioni et al., 2022). These circumstances may entail ongoing awareness of the genome diversity and the virulence alteration of GI.2 in consequence of its persistent transmission in rabbits.

In 2020, the GI.2 strain was identified in the Sichuan province in China (Hu et al., 2021), during an outbreak of the RHD, which was suspected to be the result of international import due to its high nucleotide homology with Netherlands isolates in 2016 (Qi et al., 2022). Recently, intergenotypic recombination between non-structural and structural genome segments derived from different genotypes was considered as the main mechanism of genetic evolution in Lagovirues (Mahar et al., 2021). Hence this mechanism may also be a robust driver for GI.2 variants to expand host range and adaption. Hitherto, several recombination patterns have been confirmed for GI.2 variants, including intergenotypic recombination between pathogenic GI.1b and GI.2 (e.g., GI.1bP-GI.2), between non-pathogenic RCV and GI.2 (e.g., GI.4eP-GI.2, GI.4 cP-GI.2, GI.3P-GI.2) (Lopes et al., 2017; Mahar et al., 2018; Silvério et al., 2018; Abrantes et al., 2020b). The GI.4eP-GI.2 and GI.4 cP-GI.2 variants are progressively replacing the previous parental GI.2 in a relatively short period, strengthening the inference that genome substitution on the non-structural region may accelerate the evolutionary adaption of the virus (Mahar et al., 2021) and alter its virulence (Smertina et al., 2021).

To understand the dynamics of GI.2 in domestic rabbits since its invasion into mainland China, we characterized the genome of GI.2 variants of concern and confirmed their pathogenicity alteration in present study. Here, we first described recombination events between GI.1a and GI.2 on rabbit farms in China, which generated the novel GI.1aP-GI.2 variants. The results of this study emphasize the need to implement epidemiological surveillance of *Lagoviruses* to unravel their co-circulation and evolution, in order to adapt the prevention program.

## 2. Materials and methods

### 2.1. Sample collection and molecular detection

Twenty-two rabbit liver samples were collected from twelve rabbit farms affected by RHD in Sichuan, Shandong, Anhui, and Yunnan provinces, from May 2020 to November 2022. Total RNA was extracted from the liver samples using the RNAiso plus reagent (TaKaRa, China), then the reverse transcription was performed with the PrimeScript™ RT Reagent Kit (TaKaRa, China). All the samples were confirmed as GI.2 positive by a differential Taqman RT-PCR assay as previously described (Zhou et al., 2022).

Primers targeting the RdRp-VP60 region were designed using Primer Premier 6.0 software (PREMIER Biosoft, USA) to generate a 994 bp-long amplicon by RT-PCR, then the PCR products were sequenced using ABI 3730XL platform (Sangon Biotech Co., China) for further recombination analysis. Eight pairs of primers spanning the complete GI.2 genome were used to obtain the PCR products of five representative GI.2 strains. The PCR products were purified and inserted into the pMD19-T vector (TaKaRa, China), and at least three positive clones of each fragment were submitted for nucleic acid sequencing. Information on all primers and clinical samples were listed in Supplementary Table S1, S2.

### 2.2. Histopathology and transmission electron microscopy

The gross pathological findings in the dead rabbits were recorded, and subsequent necropsies were performed according to routine procedures. The liver tissue blocks were fixed in 4% paraformaldehyde for 24 h, then paraffin-embedded, sectioned at 4 μm, and stained with hematoxylin and eosin. The histopathology of the liver section was observed under the light microscope (Leica, Germany). For the transmission electron microscopy (TEM), virus particles were purified as described before (Hu et al., 2010) with a minor modification. The infected liver tissues were homogenized and quickly frozen and thawed to release the virus particles. The virus suspension was collected after centrifugation (10,000 g, 20 min) at 4°C. Then, the supernatant was treated with 6% (w/v) polyethylene glycol (PEG 6000) and 3% (w/v) NaCl overnight at 4°C. The precipitate was resuspended in PBS after low-speed centrifugation (4,450 g, 40 min, 4°C), and then combined with a mixture of butanol and isopentanol (24:1, v/v) and stirred for 5 min. The suspension was clarified by low-speed centrifugation (430 g, 40 min, 4°C). The aqueous phase was collected and centrifuged at 15,000 g for 40 min. The supernatant was sent to the Chengdu Lilai Biomedicine experiment center for virus particle detection under the TEM (JEOL, Japan).

### 2.3. Genome alignment and phylogenetic analysis

All sequences were retrieved from the GenBank database, including the representative genomic sequences of 61 *Lagoviruses* of different genotypes (Supplementary Table S3). The complete genome sequences of SCMS-2020 (GenBank accession: OQ570964), SCNJ-2021 (GenBank accession: OQ570963), SDRZ-2021 (GenBank accession: OQ570961), SCMS-2022 (GenBank accession: OQ570960), and AHFY-2022 (GenBank accession: OQ570962) were obtained by sequence assembly. The nucleotide and amino acids identity of the RHDV strains alignment were analyzed using the MegAlign program within DNASTAR 7.0 software (DNASTAR Inc., Madison, WI, USA). The phylogenetic analysis of complete genome sequences was performed using MEGA 10 with the maximum-likelihood approach based on NSPs fragments (nt positions 10-5304), VP60 fragments (nt positions 5305-7044), and complete genome using the GTR+ G + I model. The reliability of nodes was assessed by the bootstrap resampling procedure consisting of 1,000 replicates.

### 2.4. Recombination analysis

The Recombination Detection Program 4 (RDP4, v4.24) containing seven evaluation algorithms (RDP, Bootscan, GENECONV, MaxChi, Chimera, SiScan, and 3Seq) was used to confirm the putative recombination events and precise recombination breakpoints. Recombination events were deemed significant (value of *p* ≤1× 10^−6^) when supported by at least five of the seven algorithms. SimPlot (v3.5.1, Baltimore, MD, USA) with a 200-bp window sliding along the genome (20-bp step size) was used to analyze the new variants. The recombinant *Lagoviruses* were defined using the nomenclature [RdRp genotype]P-[capsid genotype].

### 2.5. Hemagglutination and hemagglutination inhibition assays

The Hemagglutination (HA) and hemagglutination inhibition (HI) were performed as described previously (Mizoguchi et al., 2003; Song et al., 2017). For the HA, the liver tissue was homogenized on ice, then the supernatant was collected after centrifugation. Type B human red blood cells were washed in phosphate-buffered saline (PBS) and later centrifuged (280 g, 10 min) at room temperature. The RBC pellets were then resuspended and diluted in PBS (pH 7.2) to the final concentration of 1%. Then, 25 μL supernatant of liver homogenate was added into 96-well V-shaped bottom microtiter plates and two-fold serially diluted with equal volume of PBS (pH 7.2). Later, 25 μL of 1% human type B RBCs was added to each well and incubated at 25°C for 30–60 min. The HA titer was determined as the highest dilution that caused complete hemagglutination of RBCs.

For the HI, the collected sera was inactivated and pretreated with 25% kaolin (Macklin, China). Then, 25 μL of serum was added into 96-well V-shaped bottom microtiter plates and two-fold serially diluted with equal volume of PBS 25 μL of RHDV antigens (4 HAU) was added into each well and incubated at 25°C for 30–60 min. Subsequently, 25 μL of human type B RBCs were added into each well and settled at 25°C for 30–60 min. The highest dilution that caused complete inhibition was considered the hemagglutination inhibition titer. HI titer ≤2^3^ was considered as antibody negative.

### 2.6. Vaccine inoculation and lethal challenge with GI.1aP-GI.2 in experimental rabbits

Four-week-old (juvenile) and three-month-old (adult) New Zealand white rabbits were raised in the Experimental Animal Center of Huapai Biological Group Co., Ltd. (Chengdu, China). These rabbits were tested and shown to be seronegative to GI.1/GI.2 (HI titer≤ 2^3^). After adaptive feeding for one week, 60 rabbits were randomly allocated into the unvaccinated group and the vaccinated group (receiving a single dose of inactivated bivalent RHDV vaccination consisting of inactivated GI.1a and GI.2 antigen). The inactivated bivalent RHDV vaccine was prepared as follows. First, the liver homogenates were prepared from naive rabbits that died of RHD with GI.1a or GI.2 challenge. After being inactivated by formaldehyde, the liver homogenates were mixed with a 1:1 ratio of each antigen (512 HAU of each genotype). The rabbits in the vaccinated group received a subcutaneous inoculation of 1-ml laboratory-produced bivalent vaccine. To determine the virus challenge dose, we implemented the calculation of median lethal dose (LD_50_) by inoculating rabbits with a series of 10-fold dilutions of liver homogenates containing SCMS-2020 strain or SCNJ-2021 strain (5 rabbits per dilution). Then, the LD_50_ values were calculated by the Reed-Muench method (Supplementary Table S4). The LD_50_ values for SCMS-2020 strain and SCNJ-2021 strain were determined as 10^−4.68^ LD_50_ and 10^−5.5^ LD_50_ in 1-ml liver homogenates, separately. On the 14th day after vaccination, the rabbits in the vaccinated group (The antibody HI titers were between 2^7^and 2^9^) were challenged with a dose of 10,000 LD_50_ for GI.2 (SCMS-2020 strain) or 10,000 LD_50_ for GI.1aP-GI.2 (SCNJ-2021 strain), respectively. The rabbits in the unvaccinated group were also challenged with a dose of 10,000 LD_50_ for each strain. The survival time and mortality were recorded within 96 h post-infection.

## 3. Results

### 3.1. Pathological findings and virus particle identification

**Figure 1:**
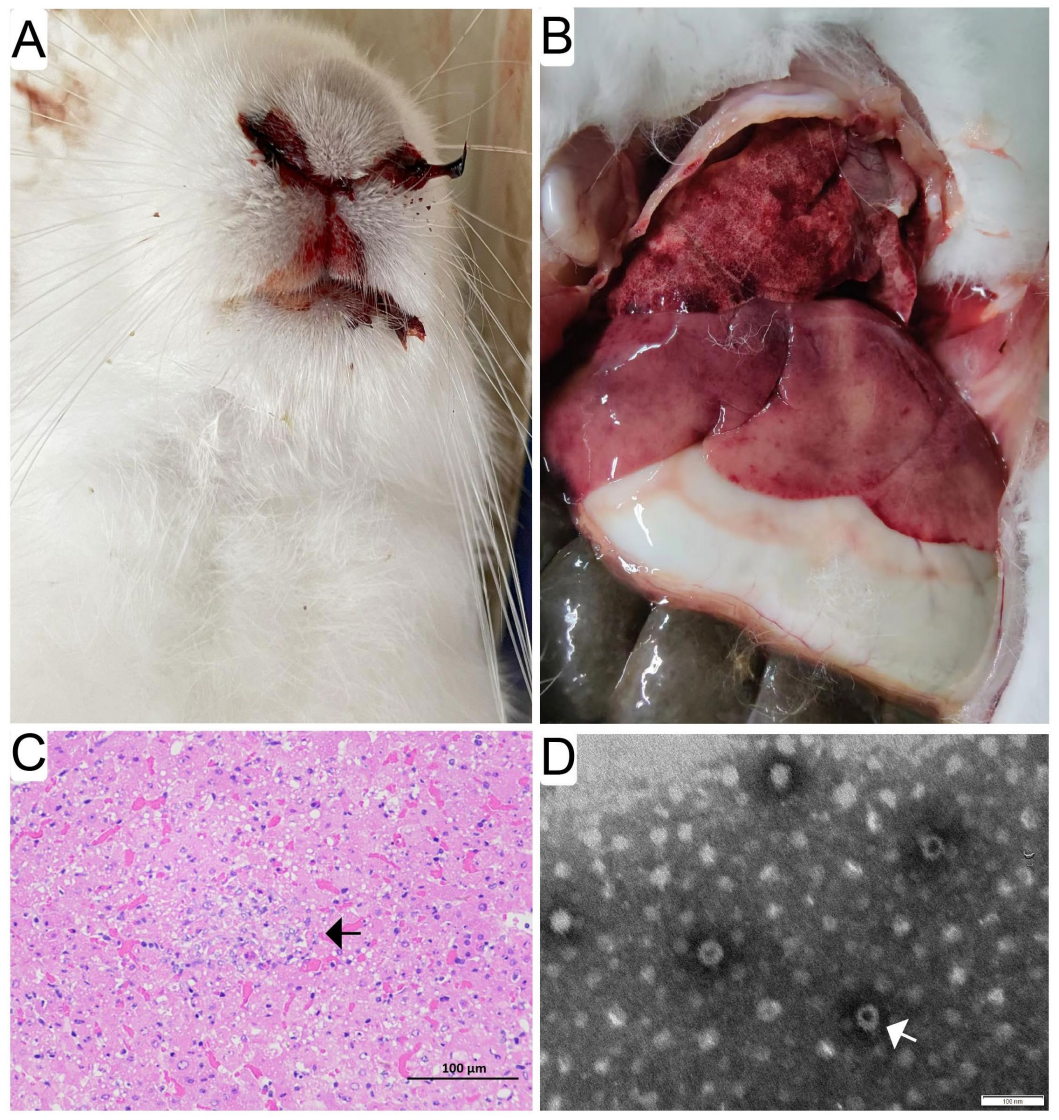
Gross pathological and histopathological findings in infected rabbits. Evident epistaxis was observed in approximate 6% of the infected rabbits undergoing acute and subacute RHD **(A)**. Enlarged, yellow-tan and mottled liver lobes were remarkable abnormal, and multifocal coagulopathy was also found in lungs and other organs **(B)** at necropsy. Significant histopathological lesions were confirmed in the liver and spleen of all animals. Evident cellular necrosis with hemorrhage appeared throughout the disarranged hepatic parenchyma, which was infiltrated by a large number of heterophils (white arrow) (bar = 100 μm) **(C)**. The purified and negatively stained virus particles were shown (white arrow) under electron micrographs (bar = 100 nm) **(D)**.

The diseased rabbits succumbed to the RHD were examined at necropsy and evaluated histologically. The results showed that epistaxis was observed in approximate 6% of the rabbits undergoing acute and subacute RHD (Figure 1A). Enlarged, yellow-tan and mottled liver lobes were remarkably abnormal, and multifocal coagulopathy were also seen in lungs and other organs (Figure 1B) at necropsy. Significant histopathological lesions were confirmed in the liver of all animals. Evident cellular necrosis with hemorrhage appeared throughout the disarranged hepatic parenchyma, which was infiltrated by a large number of heterophils (Figure 1C). In addition, the evidence of virions in infected liver tissues was confirmed by the TEM. The visible icosahedral symmetry of the virus particles, approximately 30 nm in diameter with an inner shell, were consistent with GI.1/GI.2 (Figure 1D).

### 3.2. Identification of novel GI.1aP-GI.2 variants

Twenty-two rabbit liver samples tested positive for GI.2 VP60 gene, were collected in rabbit farms from Sichuan, Anhui, Shandong, and Yunnan provinces from 2020 to 2022 during RHD outbreaks (Supplementary Table S2) and none was positive for GI.1 VP60 gene. Then, we obtained the nucleotide sequences of the RdRp-VP60 junction derived from above GI.2 isolates. The recombinant analysis of the RdRp-VP60 junctions confirmed that nine of the 22 isolates (40.9%) were presumptive recombinant GI.1aP-GI.2 strains, while the others belonged to the parental GI.2 strains (Supplementary Figure S1; Supplementary Table S2). Eight overlapping fragments of each representative strain were obtained and sequenced (Supplementary Figure S2). Nucleotide alignments of the consensus sequences confirmed that SCNJ-2021 (OQ570963), SDRZ-2021 (OQ570961), SCMS-2022 (OQ570960) and AHFY-2022 (OQ570962) were the recombinant strains belonging to the GI.1aP-GI.2 clade, whereas SCMS-2020 (OQ570964) was classified as a prototype of GI.2.

### 3.3. Phylogenetic features of the GI.aP-GI.2 variants

**Figure 2:**
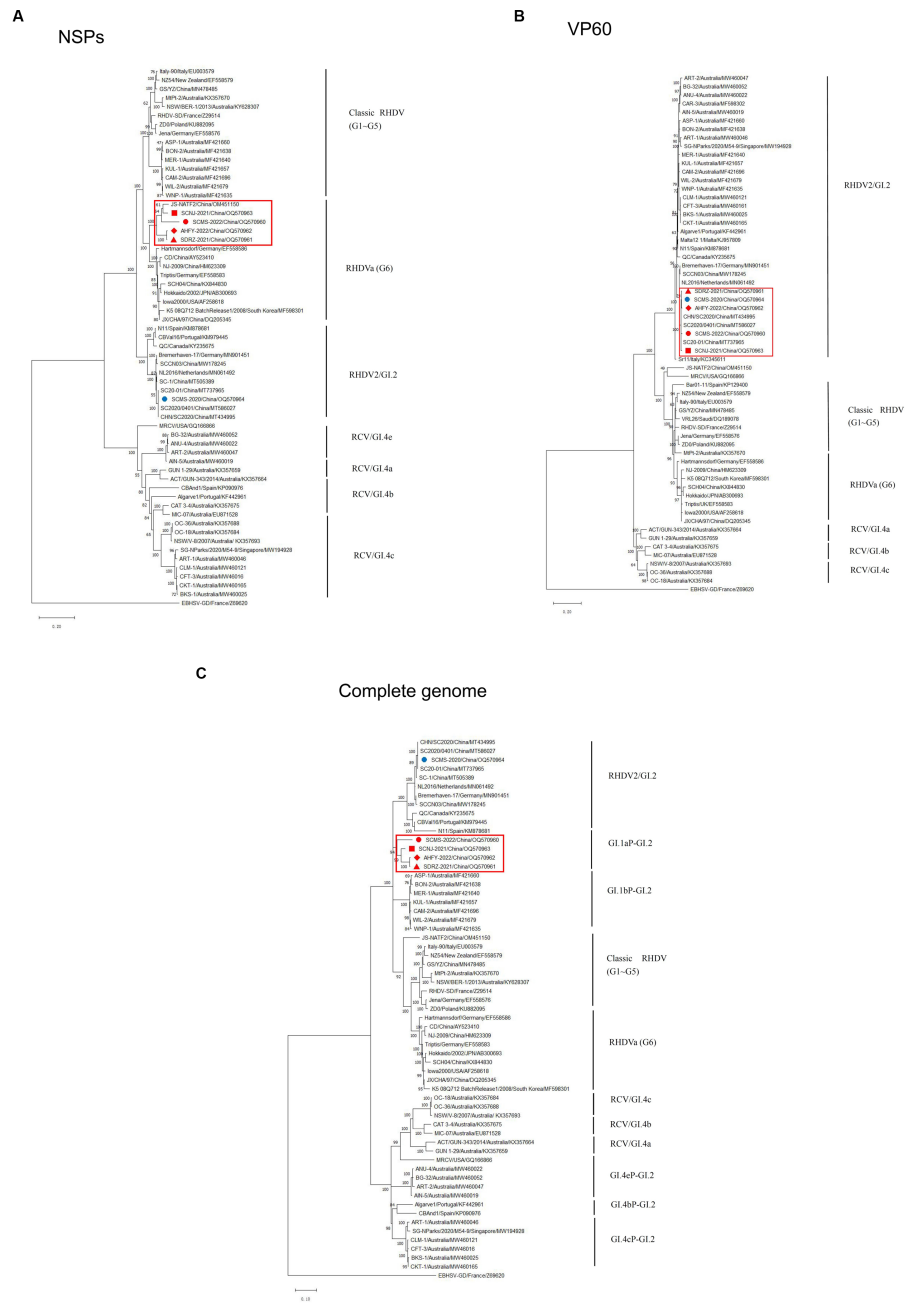
Phylogenetic trees based on NSPs **(A)**, VP60 **(B)** and full-length genome sequence **(C)** of five isolates with 56 RHDV and RCV representative strains available in GenBank. Major genetic groups (genogroups) are indicated and the five isolates in this study are labeled. The phylogenetic tree was constructed by using the MLmethod (1,000 bootstrap) in MEGA 10. Numbers along branches are bootstrap values. Scale bar indicates nucleotide substitute per site.

To unveil the genetic characterizations of these recombinant GI.2 variants, their genome sequences were comprehensively analyzed by bioinformatics tools. The ML phylogenetic trees based on the NSPs coding region (nt 10-5304, indicating the sequence used as reference of these positions), VP60 gene (nt 5305-7044, indicating the sequence used as reference of these positions), and complete genome were constructed separately. The genetic analysis based on the NSP genes revealed that SCNJ-2021, SDRZ-2021, SCMS-2022, and AHFY-2022 had 86.4-89.1% nucleotide identity and 96.0-97.8% amino acid identity with reference RHDVa strains (i.e., Triptis, Iowa2000, JX/CHA/97) and grouped into a new branch with a recently reported virus strain (JS-NATF2/China/OM451150) identified in *Oryctolagus cuniculus* (Figure 2A). However, the phylogenetic profiles based on the VP60 gene showed that these four recombinant strains closely clustered with other reference GI.2 strains from China (i.e., CHN/SC2020, SC2020/0401, SC20-01 and SC-1), and the nucleotide identity was up to 98.6-99.0% (Figure 2B). Significantly, the four variants (SCNJ-2021, SDRZ-2021, AHFY-2022, and SCMS-2022) branched into a monophyletic group showing 84.7-88.6% nucleotide identity with other representative strains based on the complete genome analysis (Figure 2C; Supplementary Tables S3, S6).

### 3.4. Recombination events of the GI.1aP-GI.2 variants

**Figure 3:**
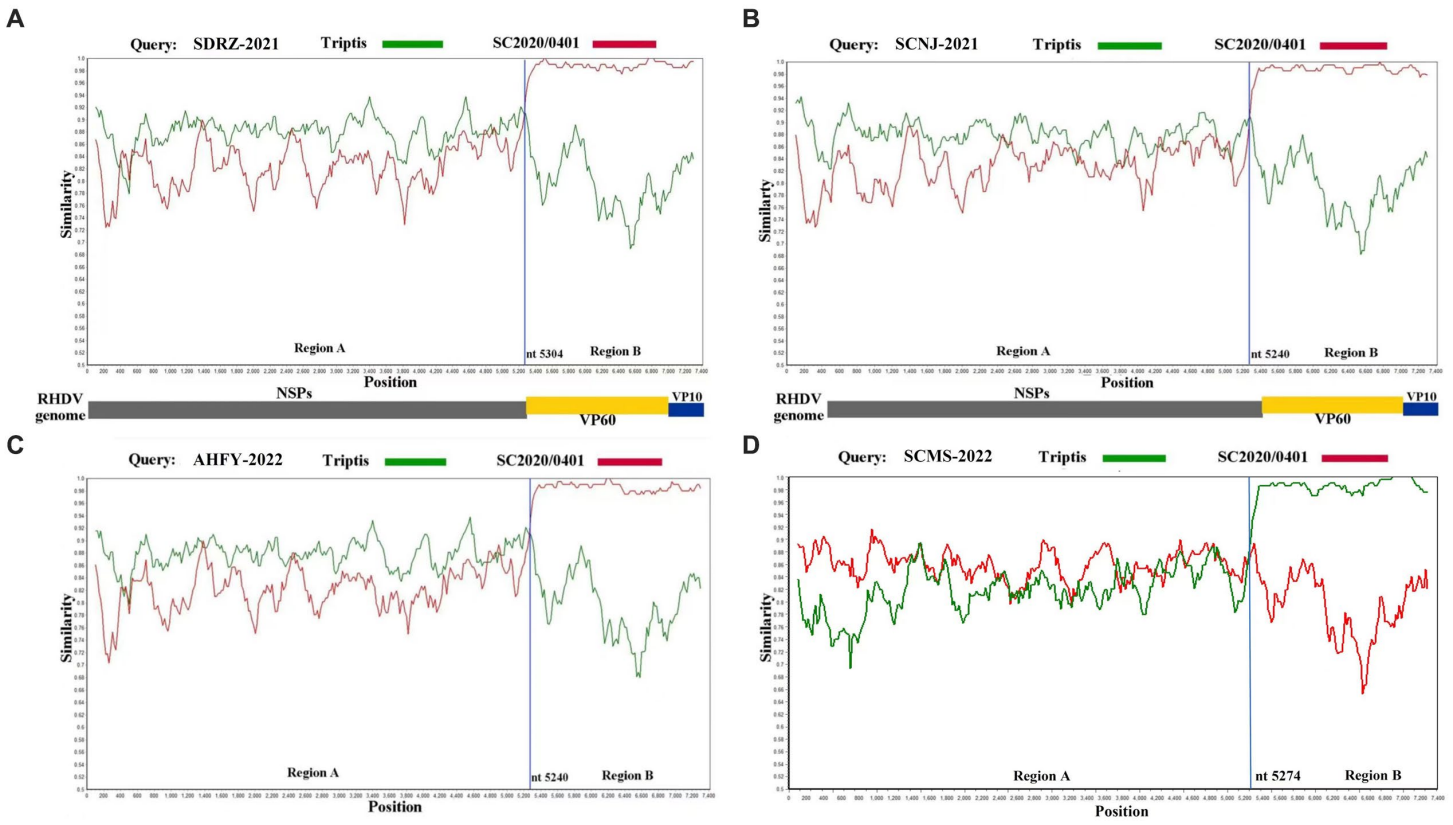
Recombination analysis of SDRZ-2021 **(A)**, SCNJ-2021 **(B)**, AHFY-2022 **(C)**, and SCMS-2022 **(D)** strains. Analysis was conducted using a sliding window of 200-bp window and a 20-bp step. The y-axis indicates the percentage similarity between the query sequence and the reference sequences. **(A)** Genome scale similarity comparisons of SDRZ-2021 (query) with Triptis (green) and SC2020/0401 (red); **(B)** genome scale similarity comparisons of SCNJ-2021 (query) with Triptis (green) and SC2020/0401 (red); **(C)** genome scale similarity comparisons of AHFY-2022 (query) with Triptis (green) and SC2020/0401 (red); **(D)** genome scale similarity comparisons of SCMS-2022 (query) with Triptis (green) and SC2020/0401 (red). The recombination breakpoints are marked at the bottom with nucleotide sites and viral genome structure referenced to SC2020/0401.

To further verify this novel recombination pattern, the recombination events of these four variants were analyzed by Recombination Detection Program 4 and Simplot software. At least five methods were used to confirm the recombination of the complete genome of SCNJ-2021, SDRZ-2021, AHFY-2022, and SCMS-2022 variants by the analysis (*p*-values of ≤1 × 10^−6^) (Supplementary Table S7). Similarity plot analysis confirmed the recombination breakpoints along the genomic RdRp-VP60 junction (nt 5240, nt 5274, or nt 5304) (Figure 3). Determined by the RDP4 software, the most likely parental variants for the four strains were pathogenic GI.1a donating the non-structural genome segment (Genbank accession EF558583) and pathogenic GI.2 donating the structural genome segment (Genbank accession MT586027).

### 3.5. Cross-protection between GI.2 and GI.1aP-GI.2 variant

In order to demonstrate whether the vaccine prepared from the prototype GI.2 strain conferred protection against the recombinant GI.2 variant, the challenge study was carried out. As expected, all unvaccinated juvenile and adult rabbits died of the challenge with the GI.2 or GI.1aP-GI.2 variant from 24 h to 96 h post-infection. However, the vaccination with GI.2 (SCMS-2020) provided complete protection against parental GI.2 infection and incomplete cross-protection against GI.1aP-GI.2 (SCNJ-2021) infection. The protection was slightly lower in vaccinated juvenile rabbits than in adult rabbits for G1.1aP-GI.2 (SCNJ-2021) variant (Table 1), and all rabbits succumbed to the virus infection experienced subacute or acute disease course. The survival time for rabbits challenged with GI.2 (SCMS-2020) or GI.1aP-GI.2 (SCNJ-2021) in both unvaccinated and vaccinated groups were not significantly different (Supplementary Table S5).

**Table 1:** Challenge results using prototype GI.2 and GI.1aP-GI.2 strain.

Challenge virus	Infection dose	Unvaccinated group^a^		Vaccinated group^a,b^	
		Juvenile rabbits	Adult rabbits	Juvenile rabbits	Adult rabbits
		Survival/Total (Survival rate)	Survival/Total (Survival rate)	Survival/Total (Survival rate)	Survival/Total (Survival rate)
SCMS-2020 (GI.2)	10,000 LD_50_	0/6 (0%)	0/6 (0%)	6/6 (100%)	6/6 (100%)
SCNJ-2021 (GI.1aP-GI.2)	10,000 LD_50_	0/6 (0%)	0/6 (0%)	4/6 (66.6%)	5/6 (83.3%)
Negative control	—	3/3 (100%)	3/3 (100%)	3/3 (100%)	3/3 (100%)

^a^GI.1/GI.2 antigens and antibodies are negative at the beginning of the trial. ^b^Vaccinated with bivalent vaccines consisting of inactivated GI.1and GI.2 antigen.

## 4. Discussion

### 4.1. Nucleotide sequencing provides insight for newly emerging GI.1aP-GI.2 variants

Since the first notice of GI.2 strains in 2010 (Le Gall-Reculé et al., 2011), the ongoing recombination events among GI.2 and other genotypes of *Lagoviruses* generated several variants that emerged as prevalent strains with wide distribution in the world. For a long time, GI.1a once had been the predominant variant in China until the GI.2 outbreak in 2020. When we investigated the presence of GI.1 and GI.2 within the scope of routine disease monitoring due to the typical symptoms and necropsy findings in dead rabbits, new GI.2 variants from suspect liver specimens were identified and further investigations were conducted to determine the genetic diversity of GI.2 genotypes. The RdRp-VP60 junction is considered as a robust recombination hotspot, therefore, nucleotide sequencing for this region is a rapid detection approach for recombination analysis. The highly intergenotypic recombination frequency (40.9%) of GI.1a and GI.2 indicates that these novel variants are likely to be the predominant strains in the following years. Although this study may underestimate the current incidence of these predominant variants in the rabbit industry in China due to the lack of submission data. The results strongly support the rapid emergence of the new epidemic variants (GI.1aP-GI.2) since the outbreak of GI.2 in China. As far as we know, this recombination pattern has never been described before.

### 4.2. Genetic recombination inferred evolutionary adaption of GI.1aP-GI.2 variants

The NSP encoding genes of *Lagoviruses* determine the potential of virus replication and immune evasion (Urakova et al., 2015; Zhu et al., 2022), which is a complex process promoting virus evolution with frequent nucleotide variation (Silvério et al., 2018; Mahar et al., 2021). In this study, the recombination analysis reveals that the GI.1aP-GI.2 variants branches into a single clade, which possess similar molecular origins of the parental GI.1a and GI.2, indicating that this new recombination event may occur in China’s RHDV strains after the outbreak of GI.2 in 2020 (Chen et al., 2022). Furthermore, the high nucleotide within these four variants also demonstrates that these novel recombinant strains have close geographical relationship. Meanwhile, the position of recombination breakpoints in these four variants is flexible, which shapes the intragenotypic diversity of GI.2 recombination pattern under the evolutionary selection. Nevertheless, the mechanism of the recombination between GI.1a and GI.2 is not fully elucidated.

It has been widely reported that exposure to prototype GI.1 and GI.2 infection in rabbits might promote robust recombination between these two genotypes in a large-scale temporal and spatial context (Silvério et al., 2018; Abrantes et al., 2020a; Al-Ebshahy et al., 2022). Similar results in rabbits and hares co-infected with RHDV2 (GI.2) and the European brown hare syndrome virus (EBHSV GII.1) were also confirmed (Le Gall-Reculé et al., 2017). Hence, intergenotypic recombination between GI.1a and GI.2 may indicate a new exaptation of GI.2 counterparts in response to co-evolutionary interaction between host and virus (Schwensow et al., 2014). This predominant recombination pattern in our findings reveals that GI.1aP-GI.2 variants possibly adapt certain population expansion strategies, which may be attributed to RdRp speed and fidelity, to gain their evolutionary advantage and persistence among GI.2 variants (Mahar et al., 2021). Additionally, the possibly antibody-mediated selection pressure to these two viruses may also favor GI.1aP-GI.2 variants to replace the parental GI.2 and to acquire strengthening adaptability (Hall et al., 2021; O'Connor et al., 2022; Patel et al., 2022). However, lack of direct evidence that host immunity contributes to GI.1aP-GI.2 recombination warrants further investigation.

### 4.3. Novel recombination might contribute to altering the virulence of GI.1aP-GI.2 variants

The impact of genetic recombination on the virulence of GI.2 variants has been investigated in previous studies (Calvete et al., 2018; Müller et al., 2021). The occurrences of broad-spectrum cross-protection among GI.1bP-GI.2, GI.4eP-GI.2, and GI.4cP-GI.2 variants, irrespectively of recombination patterns and challenge dosages, were confirmed (Calvete et al., 2018; Müller et al., 2021; O'Connor et al., 2022). Nevertheless in our study, vaccination with parental GI.2 confers incomplete cross-protection against challenge by GI.1aP-GI.2 variants in vaccinated rabbits. This suggests that the new variant can escape the host’s adaptive immunity yielded by parental GI.2 and evolve to disequilibrate the host-virus interaction (Mahar et al., 2016; Lopes et al., 2018).

Although there is no statistical difference among the survival time for rabbits in each group, the increased mortality and lower LD_50_ value for the GI.1aP-GI.2 variant still support our hypothesis that the distinctive recombination mechanisms may favor GI.1aP-GI.2 variant to acquire moderately enhanced pathogenicity. Although a large amount of nucleotide and amino acid variation emerged along the genome of recombinants, no consistent substitution sites within the non-structural or structural genes were clearly confirmed to be associated with the virulence alteration for the recombinant GI.2 variants (data not shown), which also warrants further biological study and bioinformatic research to elucidate these genetic variations. According to the biocontrol management of wild *Lagomorphs* in several countries, early exposure to non-pathogenic RCV or pathogenic GI.1 conferred partial protection to GI.2 challenge (Patel et al., 2022; Taggart et al., 2022). However, rapid emergence of recombinant variants among co-circulation strains made the wild *Lagomorphs* biocontrol program less successful than before, which also implys that undesirable genetic recombination may disable the vaccine-induced protection conferred by parent RHDV strains. As well known, VP60 protein is considered as a major antigen and virulence determinant of GI.2 (Miao et al., 2019; Liu et al., 2022). Interestingly, in a survey study, the emergence of GI.2 VP60-based recombinants were more likely to be predominant strains among circulating intergenotypic variants (Mahar et al., 2021), which suggests the competitive advantage of the GI.2 recombinants over other genotypes due to frequent amino acid substitions along the NSPs, but not their VP60 protein. In addition, the molecular disparity between GI.1aP-GI.2 and other GI.2 recombinants also implies that the NSPs may not only be relevant to the virus fitness (Mahar et al., 2021), but also be associated with the virulence alteration to a certain extent.

These findings underpin the importance of genetic variability for the rapid spread of the GI.1aP-GI.2 strains in the rabbitries in China under environmental pressure and also implicate the potential of these variants to manipulate the host’s immunity.

## 5. Conclusion

To the best of our knowledge, the new recombinant GI.1aP-GI.2 was identified in domestic rabbits in China for the first time, which expanded the knowledge on the phylodynamics and genomic diversity of GI.2 genotypes. The rapid molecular evolution and varied pathogenicity of these virus recombinants highlight the urgent need for epidemiological surveillance and for future prevention of neglected GI.2 variants.

## Data availability statement

The datasets presented in this study can be found in online repositories. The names of the repository/repositories and accession number(s) can be found in the article/Supplementary material.

## Ethics statement

The animal study was reviewed and approved by the Committee on the Ethics of Animal Experimental Center of Huapai Biological Group (permit number: 2022HPAES018).

## Author contributions

JH and YL study design. DD, YL, LS, CY, and HZ experiment implement. LZ, JY, and JH data analysis and manuscript revision. YL and JH manuscript writing. All authors have approved the manuscript.

## Funding

This research is supported by the Southwest Minzu University Research Startup Funds (Grant No. RQD2021098), Natural Science Foundation of Sichuan Province (Grant No. 2022NSFSC0081), and the Public Welfare Scientific Research Institutes Basic Research Projects (Grant No. SASA202302).

## Conflict of interest

DD was employed by Huapai Bio-engineering Group Co., Ltd.

The remaining authors declare that the research was conducted in the absence of any commercial or financial relationships that could be construed as a potential conflict of interest.

## Publisher’s note

All claims expressed in this article are solely those of the authors and do not necessarily represent those of their affiliated organizations, or those of the publisher, the editors and the reviewers. Any product that may be evaluated in this article, or claim that may be made by its manufacturer, is not guaranteed or endorsed by the publisher.
