# Supplementary material for: First report of GI.1aP-GI.2 recombinants of rabbit hemorrhagic disease virus in domestic rabbits in China
Source: Front Microbiol. 2023 Jul 14;14:1188380. doi: 10.3389/fmicb.2023.1188380 (PMC10382137; doi:10.3389/fmicb.2023.1188380)
Supplement: Supplementary file 1 [file Data_Sheet_1.docx]

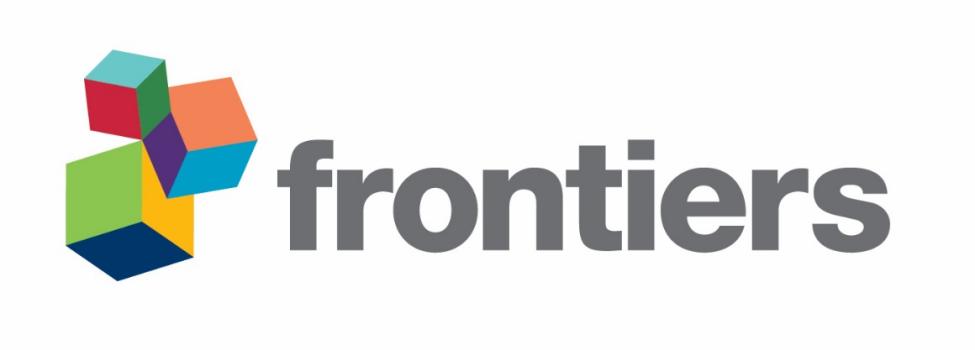


Supplementary Material

**First report of GI.1aP-GI.2 recombinants of rabbit hemorrhagic disease virus in domestic rabbits in China**

*** Correspondence:**Corresponding Author
Jian Huang, hjvet03@sin.cn

**Supplementary Table S1** Primers for genome amplification, GI.2 molecular detection and recombination analysis

| **Primers Sequence** | **Position** | **Size** | **Source** |
| --- | --- | --- | --- |
| RHDV2-A-F 5’-GTGAAAGTTATGGCGGCTATG-3’  RHDV2-A-R 5’-CGGCCGCAGTCGTGTATGTA-3’  RHDV2-B-F 5’-TCCTGGACCTCAGGGAAGAGA-3’  RHDV2-B-R 5’-GCCATTTTCACAACTGTCAT-3’  RHDV2-C-F 5’-ATGGCTTTTGTCTTTTCCAC-3’  RHDV2-C-R 5’-AGGACGTCAACACCTTGGTCA-3’  RHDV2-D-F 5’-GGTGTGTGAACATGACAAACCA-3’  RHDV2-D-R 5’-CATACAACGGGAGCCCGCAGTC-3’  RHDV2-E-F 5’-AACACCCACACCGCCCGGTC-3’  RHDV2-E-R 5’-CGTCAGGTACTACCGAGACGA-3’  RHDV2-F-F 5’-GTGATGACGGTGTGTACGCCATG-3’  RHDV2-F-R 5’-GGCACTAGCATACCAAAGCTC-3’  RHDV2-G-F 5’-GATAACAGATGGAATGGTGAGA-3’  RHDV2-G-R 5’-GCACCTGCAAGTCCAAGTCCA-3’  RHDV2-H-F 5’-GGCTTCATGGAACTTGGCTTG-3’  RHDV2-H-R 5’-GACTGACTGCCATGGCCGGCGCTAGC-3’  RHDV2-H-R2 5’-GACTGACTGCCATGGCCGGCGCTAGCTT  TTTTTTTTTTTTTTTTTTTTTTT-3’  Rec-detect F 5’-GAYGACGGCGTGTATGCCATG-3’  Rec-detect R 5’-CATGGTGATGGTRACAGGTTCGA-3’ | 1-21 nt  316-335 nt  80-100 nt  1336-1355 nt  1153-1172 nt  2823-2843 nt  2401-2423 nt  3640-3661 nt  3406-3425 nt  5267-5287 nt  4820-4842 nt  6244-6264 nt  6064-6085 nt  7051-7071 nt  6868-6888 nt  4821-4842 nt  5792-5814 nt | 336 bp  1276 bp  1691 bp  1261 bp  1882 bp  1445 bp  1008 bp  601 bp  994 bp | This study  Miao et al., 2019  This study  This study  This study  This study  This study  This study  Miao et al., 2019  This study |
| RHDV-F1 5’-TGGARMTWGGYTTRAGTGTDGAYG -3’  RHDV-R1 5’-CAGACATAAGAAAARCCATTGGYTG -3’  RHDV-Probe 5’-FAM-TGAYTGAACTCATTGAYGTACGCCC-BHQ1-3’  RHDV2 - Probe 5’-VIC-TGTCAGAMCTTGTTGACATCCGCC-BHQ2-3’ | 1571-1594 nt  1715-1739 nt  1640-1664 nt  1640-1663 nt | 169 bp | Zhou et al., 2022 |


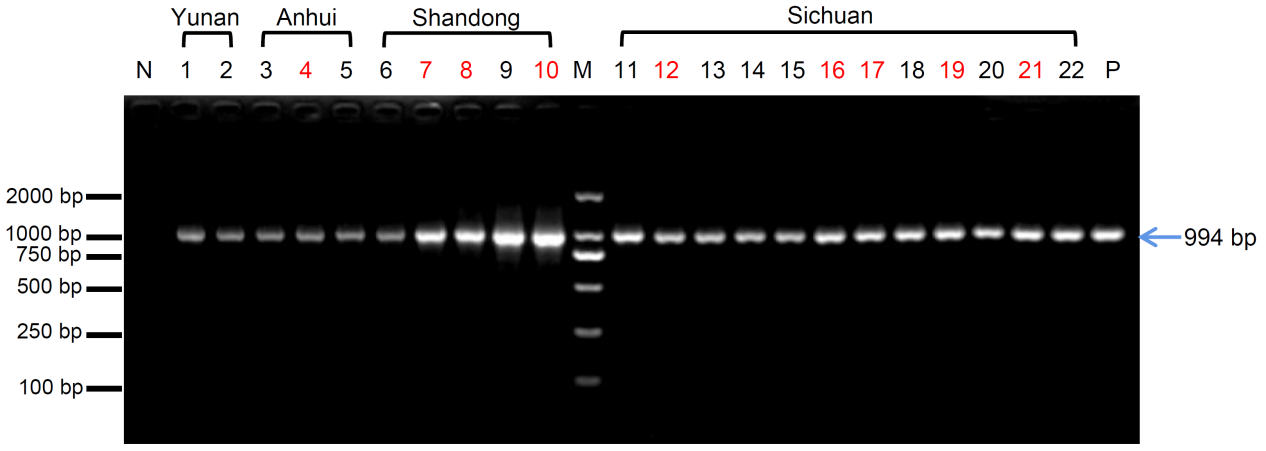


**Supplementary Figure S1** Agarose gel electrophoresis of the amplicons (994 bp) of RdRp-VP60 junction from twenty-two collected liver specimens. Nine amplicons were further confirmed as GI.1aP-GI.2 recombinants by sequencing (red numbers). M-molecular weight, N-negative control, P-positive control.


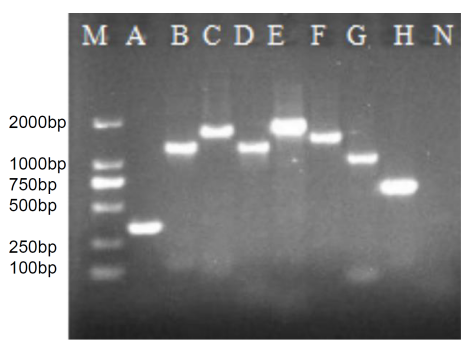


**Supplementary Figure S2** Agarose gel electrophoresis of eight amplicons corresponding to overlapping genomic fragments of the recombinant strain SDRZ-2021(OQ570961) (A,B,C,D,E,F,G,H). M-molecular weight, N-negative control.

| **Supplementary Table S2** Detailed information for clinical specimens. | | | | | | | | |
| --- | --- | --- | --- | --- | --- | --- | --- | --- |
| Number | Name | Organ tested | Host | Region | Date of collection | Genotype according to SP encoding genes | Recombinant Lineage | Mean value of Cq |
|  |  |  |  |  |  |  |  |  |
|  |  |  |  |  |  |  |  |  |
| 1 | YN-1 | Liver | Domestic Rabbit | Yunnan | 2021.12.05 | GI.2 | No | 23.01 |
| 2 | YN-2 | Liver | Domestic Rabbit | Yunnan | 2022.01.16 | GI.2 | No | 22.72 |
| 3 | AH-1 | Liver | Domestic Rabbit | Anhui | 2022.02.15 | GI.2 | No | 22.26 |
| 4 | AHFY-2022 | Liver | Domestic Rabbit | Anhui | 2022.05.03 | GI.2 | GI.1aP-GI.2 | 22.12 |
| 5 | AH-3 | Liver | Domestic Rabbit | Anhui | 2022.11.10 | GI.2 | No | 21.82 |
| 6 | SD-2 | Liver | Domestic Rabbit | Shandong | 2021.12.01 | GI.2 | No | 21.54 |
| 7 | SDRZ-2021 | Liver | Domestic Rabbit | Shandong | 2021.10.25 | GI.2 | GI.1aP-GI.2 | 17.58 |
| 8 | SD-3 | Liver | Domestic Rabbit | Shandong | 2022.08.01 | GI.2 | GI.1aP-GI.2 | 17.73 |
| 9 | SD-4 | Liver | Domestic Rabbit | Shandong | 2022.08.13 | GI.2 | No | 17.5 |
| 10 | SD-5 | Liver | Domestic Rabbit | Shandong | 2022.11.17 | GI.2 | GI.1aP-GI.2 | 16.4 |
| 11 | SCMS-2020 | Liver | Domestic Rabbit | Sichuan | 2020.05.22 | GI.2 | No | 18.27 |
| 12 | SC-4 | Liver | Domestic Rabbit | Sichuan | 2021.10.27 | GI.2 | GI.1aP-GI.2 | 21.32 |
| 13 | SCDY-1 | Liver | Domestic Rabbit | Sichuan | 2021.12.20 | GI.2 | No | 21.04 |
| 14 | SCDY-2 | Liver | Domestic Rabbit | Sichuan | 2022.01.16 | GI.2 | No | 20.63 |
| 15 | SCZG-1 | Liver | Domestic Rabbit | Sichuan | 2021.12.25 | GI.2 | No | 20.5 |
| 16 | SCNJ-2021 | Liver | Domestic Rabbit | Sichuan | 2021.11.06 | GI.2 | GI.1aP-GI.2 | 19.87 |
| 17 | SCZG-2 | Liver | Domestic Rabbit | Sichuan | 2022.01.06 | GI.2 | GI.1aP-GI.2 | 19.69 |
| 18 | SCMY-1 | Liver | Domestic Rabbit | Sichuan | 2022.03.29 | GI.2 | No | 19.6 |
| 19 | SCLS-2 | Liver | Domestic Rabbit | Sichuan | 2022.04.10 | GI.2 | GI.1aP-GI.2 | 19.3 |
| 20 | SCMY-2 | Liver | Domestic Rabbit | Sichuan | 2022.08.17 | GI.2 | No | 18.27 |
| 21 | SCMS-2022 | Liver | Domestic Rabbit | Sichuan | 2022.01.05 | GI.2 | GI.1aP-GI.2 | 17.92 |
| 22 | SCDY-3 | Liver | Domestic Rabbit | Sichuan | 2022.07.03 | GI.2 | No | 17.79 |

| **Supplementary Table S3** Sequences retrieved from GenBank database. | | | | | |
| --- | --- | --- | --- | --- | --- |
| **Strain Name** | **GenBank Accession Number** | **Country** | **Year of Isolation** | **Genotype** | **Recombinant Lineage** |
| AIN_5 | MW460019 | Australia | 2017 | GI.2 | GI.4eP-GI.2 |
| ANU_4 | MW460022 | Australia | 2018 | GI.2 | GI.4eP-GI.2 |
| ART_2 | MW460047 | Australia | 2019 | GI.2 | GI.4eP-GI.2 |
| BG_32 | MW460052 | Australia | 2018 | GI.2 | GI.4eP-GI.2 |
| CAR_3 | MF598302 | Australia | 2016 | GI.2 | GI.4eP-GI.2 |
| BKS_1 | MW460025 | Australia | 2018 | GI.2 | GI.4cP-GI.2 |
| CFT_3 | MW460161 | Australia | 2018 | GI.2 | GI.4cP-GI.2 |
| CKT_1 | MW460165 | Australia | 2018 | GI.2 | GI.4cP-GI.2 |
| CLM_1 | MW460121 | Australia | 2018 | GI.2 | GI.4cP-GI.2 |
| ART_1 | MW460046 | Australia | 2019 | GI.2 | GI.4cP-GI.2 |
| SG-NParks | MW194928 | Singapore | 2020 | GI.2 | GI.4cP-GI.2 |
| Algarve1 | KF442961 | Portugal | 2013 | GI.2 | GI.4bP-GI.2 |
| CBAnd1 | KP090976 | Spain | 2012 | GI.2 | GI.4bP-GI.2 |
| MIC_07 | NC_011704 | Australia | 2007 | GI.4b | No |
| CAT_3_4 | KX357675 | Australia | 2007 | GI.4b | No |
| BON_2 | MF421638 | Australia | 2016 | GI.2 | GI.1bP-GI.2 |
| Mer_1 | MF421640 | Australia | 2015 | GI.2 | GI.1bP-GI.2 |
| WNP_1 | MF421635 | Australia | 2016 | GI.2 | GI.1bP-GI.2 |
| KUL_1 | MF421657 | Australia | 2016 | GI.2 | GI.1bP-GI.2 |
| WIL-2 | MF421679 | Australia | 2015 | GI.2 | GI.1bP-GI.2 |
| CAM-2 | MF421696 | Australia | 2016 | GI.2 | GI.1bP-GI.2 |
| ASP-1 | MF421660 | Australia | 2016 | GI.2 | GI.1bP-GI.2 |
| GUN_1_29 | KX357659 | Australia | 2008 | GI.4a | No |
| GUN_343 | KX357664 | Australia | 2014 | GI.4a | No |
| OC_18 | KX357684 | Australia | 2007 | GI.4c | No |
| OC_36 | KX357688 | Australia | 2007 | GI.4c | No |
| V_8 | KX357693 | Australia | 2007 | GI.4c | No |
| SCMS-2022 | OQ570960 | China | 2022 | GI.2 | GI.1aP-GI.2 |
| SCNJ-2021 | OQ570963 | China | 2021 | GI.2 | GI.1aP-GI.2 |
| SDRZ-2021 | OQ570961 | China | 2021 | GI.2 | GI.1aP-GI.2 |
| AHFY-2022 | OQ570962 | China | 2022 | GI.2 | GI.1aP-GI.2 |
| SCMS-2020 | OQ570964 | China | 2020 | GI.2 | No |
| CBVal16 | KM979445 | Portugal | 2012 | GI.2 | No |
| N11 | KM878681 | Spain | 2011 | GI.2 | No |
| QC | KY235675 | Canada | 2016 | GI.2 | No |
| Bremerhaven-17 | MN901451 | Germany | 2017 | GI.2 | No |
| NL2016 | MN061492 | Netherlands | 2016 | GI.2 | No |
| SC-1 | MT505389 | China | 2020 | GI.2 | No |
| SC20-01 | MT737965 | China | 2020 | GI.2 | No |
| SC2020/0401 | MT586027 | China | 2020 | GI.2 | No |
| SCCN03 | MW178245 | China | 2020 | GI.2 | No |
| CHN/SC2020 | MT434995 | China | 2020 | GI.2 | No |
| Triptis | EF558583 | Germany | 2008 | GI.1a | No |
| Hartsmanndorf | EF558586 | Germany | 2007 | GI.1a | No |
| JX/CHA97 | DQ205345 | China | 1997 | GI.1a | No |
| RHDV-CD | AY523410 | China | 2004 | GI.1a | No |
| NJ-2009 | HM623309 | China | 2009 | GI.1a | No |
| Hokkaido | AB300693 | Japan | 2002 | GI.1a | No |
| lowa200 | AF258618 | USA | 2000 | GI.1a | No |
| K5_08Q712_BatchRelease1/2008 | MF598301 | South Korea | 2008 | GI.1a | No |
| SCH04 | KX844830 | China | 2004 | GI.1a | No |
| JS-NATF2 | OM451150 | China | 2020 | GI.4- like | G1.1aP-GI.4 like |
| MRCV | GQ166866 | USA | 2001 | G1.4- like | No |
| RHDV-SD | Z29514 | French | 1993 | classical GI.1 | No |
| NZ54 | EF558579 | New Zealand | 2007 | classical GI.1 | No |
| Italy-90 | EU003579 | Italy | 1990 | classical GI.1 | No |
| GS/YZ | MN478485 | China | 2011 | classical GI.1 | No |
| NSW/BER-1 | KY628307 | Australia | 2013 | classical GI.1 | No |
| ACT/MtPt-2 | KX357670 | Australia | 2010 | classical GI.1 | No |
| Jena | EF558576 | Germany | 2007 | classical GI.1 | No |
| ZD0 | KU882095 | Poland | 2000 | classical GI.1 | No |
| EBHSV-GD | Z69620 | France | 1996 | Calicivirus | No |

| **Supplementary Table S4** Calculation of LD_50_ values for SCMS-2020 strain and SCNJ-2021 strain by the Reed-Muench method | | | | | | | |
| --- | --- | --- | --- | --- | --- | --- | --- |
| Challenge Virus | Virus  Dilution | Died | Survived | Cumulative values | | | LD_50_ value |
|  |  |  |  | Cumulative  mortality | Cumulative  survived | Ratio  (%) |  |
| SCMS-2020 | 10^-3^ | 5 | 0 | 11 | 0 | 100 | 10^-4.68^ |
|  | 10^-4^ | 4 | 1 | 6 | 1 | 85.7 |  |
|  | 10^-5^ | 2 | 3 | 2 | 4 | 33.3 |  |
|  | 10^-6^ | 0 | 5 | 0 | 9 | 0 |  |
| SCNJ-2021 | 10^-3^ | 5 | 0 | 15 | 0 | 100 | 10^-5.5^ |
|  | 10^-4^ | 5 | 0 | 10 | 0 | 100 |  |
|  | 10^-5^ | 3 | 2 | 5 | 2 | 71.4 |  |
|  | 10^-6^ | 2 | 3 | 2 | 5 | 28.6 |  |

Reed-Muench method: Proportionate distance (PD) = [(% cumulative mortality at the next dilution above 50%)-50%]/[(% cumulative mortality at the next dilution above 50%)-(% cumulative mortality at the next dilution below 50%)]. Next, the 50% endpoint dilution was further calculated as follows: Negative logarithm of LD_50_ = (negative logarithm of the next dilution above 50% mortality + PD)×dilution factor.

**Supplementary Table S5**  Survival time of rabbits in unvaccinated and vaccinated group within time intervals post-infection.

| Challenge virus | Unvaccinated group | Time intervals post-infection | | | Death/Total  (Mortality) |
| --- | --- | --- | --- | --- | --- |
|  |  | ~48 h | ~72 h | ~96 h |  |
|  |  | Death/Total  (Mortality) | Death/Total  (Mortality) | Death/Total  (Mortality) |  |
| SCMS-2020  (GI.2) | Juvenile rabbits | 3/6 (50%) | 3/6 (50%) | _ | 6/6 (100%) |
|  | Adult rabbits | 2/6 (33.3%) | 4/6 (67.7%) | _ | 6/6 (100%) |
| SCNJ-2021  (G1.1aP-GI.2) | Juvenile rabbits | 3/6 (50%) | 3/6 (50%) | _ | 6/6 (100%) |
|  | Adult rabbits | 3/6 (50%) | 3/6 (50%) | _ | 6/6 (100%) |
| Negative control | Juvenile rabbits | 0 | 0 | 0 | 0/6 (0%) |
|  | Adult rabbits | 0 | 0 | 0 | 0/6 (0%) |

| Challenge virus | Vaccinated group | Time intervals post-infection | | | Death/Total （Motality） |
| --- | --- | --- | --- | --- | --- |
|  |  | ~48 h | ~72 h | ~96 h |  |
|  |  | Death/Total  (Mortality) | Death/Total  (Mortality) | Death/Total  (Mortality) |  |
| SCMS-2020  (GI.2) | Juvenile rabbits | 0/6 (0%) | 0/6 (0%) | 0/6 (0%) | 0/6 (0%) |
|  | Adult rabbits | 0/6 (0%) | 0/6 (0%) | 0/6 (0%) | 0/6 (0%) |
| SCNJ-2021  (G1.1aP-GI.2) | Juvenile rabbits | 0/6 (0%) | 1/6 (16.7%) | 1/6 (16.7%) | 2/6 (33.4%) |
|  | Adult rabbits | 0/6 (0%) | 0/6 (0%) | 1/6 (16.7%) | 1/6 (16.7%) |
| Negative control | Juvenile rabbits | 0/6 (0%) | 0/6 (0%) | 0/6 (0%) | 0/6 (0%) |
|  | Adult rabbits | 0/6 (0%) | 0/6 (0%) | 0/6 (0%) | 0/6 (0%) |

**Supplementary Table S6** Nucleotide and amino acid identities of genome regions of SDRZ-2021, SCNJ-2021, SCMS-2022 and AHFY-2022 compared with other Classic RHDV(GI.1), RHDVa (GI.1a) and RHDV2 (GI.2) genotypes.

| SDRZ-2021 | RHDV-SD | Jena | NZ54 | SCH04 | JX/CHA/97 | Iowa2000 | Triptis | NL2016 | N11 | SC2020/0401 | CHN/SC2020 |
| --- | --- | --- | --- | --- | --- | --- | --- | --- | --- | --- | --- |
|  | Classical RHDV | | | RHDVa | | | | RHDV2/GI.2 | | | |
|  | Pairwise % Identity to SDRZ-2021 (nt/aa) | | | | | | | | |  |  |
| Complete genome | 85.4 | 85.6 | 85 | 86.3 | 86.8 | 86.9 | 87.1 | 88.3 | 88.1 | 88.4 | 88.4 |
| NSPs  (nt 10-5304) | 86.8/96.5 | 86.7/96.5 | 86/96.2 | 88.1/97.0 | 88.6/97.1 | 88.7/97.7 | 89.1/97.5 | 84.7/96.0 | 85.1/95.9 | 84.4/95.7 | 84.4/95.6 |
| VP60  (nt 5305-7044) | 81.6/87.9 | 82.3/87.6 | 82.0/87.9 | 81.1/87.7 | 82.0/88.3 | 82.0/88.3 | 82.0/88.3 | 97.8/99.1 | 95.8/97.1 | 98.9/99.5 | 98.9/99.3 |
| VP10  (nt 7025-7378) | 84.7/82.9 | 85.8/86.3 | 85.8/83.7 | 85.6/84.6 | 85.9/83.7 | 85/83.7 | 84.7/82.9 | 96.6/100 | 96/99.1 | 98.9/100 | 98.9/100 |

| SCNJ-2021 | RHDV-SD | Jena | NZ54 | SCH04 | JX/CHA/97 | Iowa2000 | Triptis | NL2016 | N11 | SC2020/0401 | CHN/SC2020 |
| --- | --- | --- | --- | --- | --- | --- | --- | --- | --- | --- | --- |
|  | Classical RHDV | | | RHDVa | | | | RHDV2/GI.2 | | | |
|  | Pairwise % Identity to SCNJ-2021 (nt/aa) | | | | | | | | |  |  |
| Complete genome | 85.4 | 85.9 | 85.1 | 86.1 | 86.7 | 86.9 | 87.1 | 88.6 | 87.8 | 88.6 | 88.6 |
| NSPs  (nt 10-5304) | 86.7/96.8 | 87.0/96.6 | 86.3/96.1 | 87.2/96.9 | 87.8/96.9 | 88.1/97.5 | 88.4/97.4 | 85.1/95.8 | 84.8/95.8 | 84.6/95.4 | 84.6/95.3 |
| VP60  (nt 5305-7044) | 81.6/88.3 | 82.5/88.3 | 82.0/88.6 | 80.9/87.8 | 81.8/88.4 | 81.8/88.4 | 81.8/88.4 | 97.9/99.7 | 95.6/97.2 | 99.0/99.3 | 98.9/99.3 |
| VP10  (nt 7025-7378) | 85 /82.9 | 86.6 /86.3 | 86.1 /83.7 | 85.9 /84.6 | 86.2 /83.7 | 85.3 /83.7 | 85 /82.9 | 96.9 /100 | 96.9 /99.1 | 98.6 /100 | 98.6 /100 |

| SCMS-2022 | RHDV-SD | Jena | NZ54 | SCH04 | JX/CHA/97 | Iowa2000 | Triptis | NL2016 | N11 | SC2020/0401 | CHN/SC2020 |
| --- | --- | --- | --- | --- | --- | --- | --- | --- | --- | --- | --- |
|  | Classical RHDV | | | RHDVa | | | | RHDV2/GI.2 | | | |
|  | Pairwise % Identity to SCMS-2022 (nt/aa) | | | | | | | | |  |  |
| Complete genome | 84.7 | 85.3 | 84.7 | 85.1 | 85.4 | 85.6 | 85.7 | 87.9 | 87.1 | 88 | 88 |
| NSPs  (nt 10-5304) | 85.8/96 | 86.1/95.8 | 85.6/95.3 | 86.4/96.0 | 86.6/96 | 86.9/96.8 | 87.2/96.6 | 84.2/94.7 | 83.8/94.5 | 83.9/94.3 | 83.9/94.2 |
| VP60  (nt 5305-7044) | 81.8/88.4 | 82.6/88.4 | 82.1/88.8 | 81.1/87.9 | 82.0/88.6 | 81.7/88.6 | 81.7/88.6 | 97.6/99.3 | 95.4/97.6 | 98.6/99.3 | 98.6/99.1 |
| VP10  (nt 7025-7378) | 85/82.0 | 86.6/85.5 | 86.1/82.9 | 85.9/83.7 | 86.2/82.9 | 85.3/82.9 | 85/82.0 | 96.9/97.4 | 96.3/96.6 | 98.6/97.4 | 98.6/97.4 |

| AHFY-2022 | RHDV-SD | Jena | NZ54 | SCH04 | JX/CHA/97 | Iowa2000 | Triptis | NL2016 | N11 | SC2020/0401 | CHN/SC2020 |
| --- | --- | --- | --- | --- | --- | --- | --- | --- | --- | --- | --- |
|  | Classical RHDV | | | RHDVa | | | | RHDV2/GI.2 | | | |
|  | Pairwise % Identity to AHFY-2022 (nt/aa) | | | | | | | | |  |  |
| Complete genome | 85.3 | 85.6 | 85.1 | 86.2 | 86.7 | 86.8 | 87 | 88.2 | 88.0 | 88.3 | 88.2 |
| NSPs  (nt 10-5304) | 86.7/96.9 | 86.6/96.8 | 86.3/96.5 | 88.0/97.1 | 88.5/97.2 | 88.7/97.8 | 88.9/97.7 | 84.6/96.1 | 84.9/96.0 | 84.2/95.6 | 84.2/97.1 |
| VP60  (nt 5305-7044) | 81.4/87.7 | 82.5/87.7 | 81.9/88.1 | 81.0/87.6 | 81.9/88.1 | 81.9/88.1 | 81.9/88.1 | 97.8/99.3 | 95.8/97.2 | 98.7/99.1 | 98.7/99.3 |
| VP10  (nt 7025-7378) | 84.7/82.9 | 86.6/86.3 | 86.1/83.7 | 85.6/84.6 | 85.3/83.7 | 85/83.7 | 84.7/82.9 | 96/100 | 95.7/99.1 | 98.3/100 | 98.3/100 |

| **Supplementary Table 7** Recombination analysis of SCNJ-2021, SDRZ-2021, AHFY-2022, and SCMS-2022 | | | | | | | | | | |
| --- | --- | --- | --- | --- | --- | --- | --- | --- | --- | --- |
| Recombinant Strain | Breakpoint | Parental Sequence | | Detection Methods (p-Value) | | | | | | |
|  | | Minor | Major | RDP | GENECONV | BootScan | MaxChi | Chimaera | SiScan | 3Seq |
| SCNJ-2021 | 5240 | SC2020/0401 | Triptis | 1.237×10^-92^ | 1.215×10^-92^ | 2.113×10^-102^ | 1.922×10^-26^ | 2.371×10^-32^ | 1.232×10^-56^ | 2.220×10^-15^ |
| SDRZ-2021 | 5304 | SC2020/0401 | Triptis | 1.237×10^-92^ | 1.215×10^-92^ | 2.113×10^-102^ | 1.922×10^-26^ | 2.371×10^-32^ | 1.232×10^-56^ | 2.220×10^-15^ |
| AHFY-2022 | 5240 | SC2020/0401 | Triptis | 1.237×10^-92^ | 1.215×10^-92^ | 2.113×10^-102^ | 1.922×10^-26^ | 2.371×10^-32^ | 1.232×10^-56^ | 2.220×10^-15^ |
| SCMS-2022 | 5274 | SC2020/0401 | Triptis | 1.33×10^-87^ | 4.059×10^-90^ | 1.380×10^-96^ | 4.696×10^-37^ | 1.134×10^-33^ | NS | 1.110×10^-16^ |
